# Predicting the success of multimodal rehabilitation in chronic ankle instability based on patient-reported outcomes

**DOI:** 10.1186/s12891-022-05676-0

**Published:** 2022-07-25

**Authors:** Ran Zhang, Qiushi Qi, Weiqun Song, Yaping Chen

**Affiliations:** 1 Department of Rehabilitation, Xuanwu Hospital, Capital Medical University, 45 Changchunjie, Beijing, 100054 China; 2 Department of Rehabilitation, Beijing Tongren Hospital, Capital Medical University, 1 Dongjiaominxiang, Beijing, 100730 China

**Keywords:** Chronic ankle instability, Clinical prediction rules, Therapeutic intervention

## Abstract

**Background:**

The aim of this study was to identify potential indicators to predict the success of multimodal rehabilitation in chronic ankle instability (CAI) patients based on patient-reported outcomes.

**Methods:**

Sixty patients with self-reported CAI participated. Their demographic information, injury history, and symptoms were recorded. Physical examinations and dynamic posture control tests were performed. The participants underwent sixteen 30-min treatment sessions of multimodal rehabilitation over 8 weeks. Fifty-one patients (85.0%) were available for follow-up after 8 weeks of the intervention. Treatment success was defined based on the participants’ perceived recovery using the global rating of change (GRC). Potential predictor variables were entered into a stepwise logistic regression model to identify variables for the prediction of treatment success.

**Results:**

Forty of 51 participants (78.4%) were considered to have a successful outcome. Of the variables assessed, time since last sprain ≤ 8 months was a predictor of treatment success (*p* < 0.05). If a patient met the criteria, there was an 88.03% probability of successful multimodal rehabilitation.

**Conclusion:**

A time since the last sprain ≤ 8 months may predict successful patient-reported outcomes after multimodal rehabilitation in CAI patients.

**Level of evidence:**

Prospective study, Level 2.

**Supplementary Information:**

The online version contains supplementary material available at 10.1186/s12891-022-05676-0.

## Background

The most common foot–ankle and sports injury for which people seek medical treatment is ankle sprains, with a combined prevalence of 11.88% in individuals with lateral ankle sprain (LAS) among the general population [1]. The incidence of LAS may be much higher than that reported since fewer than 50% of individuals who sustain LAS seek medical attention [2]. Patients with chronic ankle instability (CAI) show decreased perceived ankle and knee joint health, more ankle pain and disability [3], damage to ankle ligaments and cartilage, and the potential to develop ankle osteoarthritis [4]. The pathology of CAI is complex and cannot be simply explained by mechanical instability and functional instability [5]. After a primary lateral ankle sprain, some patients show ongoing symptoms of pain, weakness, reduced range of motion (ROM), diminished self-reported function and recurrent ankle sprains for more than 1 year [6].

 Patients with ankle instability should first be treated nonoperatively [7]. Many previous studies have explored single or complex treatments [8, 9]. CAI patients exhibit several characteristics, such as decreased ROM, decreased strength, impaired neuromuscular control, and altered functional movement patterns [10]. For patients with CAI, multimodal rehabilitation protocols targeting four main areas of rehabilitation are recommended (ROM, balance, strength, and overall activity) [5, 8, 9, 11, 12]. Most CAI patients respond well to conservative treatment, but some patients complain that there is no change in pain or perceived ankle function [13]. Identifying whether a patient could have a good response after a rehabilitation protocol could help the patient and therapist make the right choice.

 Clinicians can use clinical prediction rules (CPRs) as a practical, evidence-based tool to help them identify patients whose condition is more likely to improve after a specific intervention [14]. CPRs have been reported to treat nonspecific low back pain [15], patellofemoral pain with orthotics [16], acute ankle sprains with manipulative therapy [13], CAI with manual therapy, such as ankle joint mobilization, plantar massage, or calf stretching [2], CAI with balance training [14], and CAI with sensory-targeted ankle rehabilitation strategies (STARS), and manual therapy, such as ankle joint mobilization, plantar massage, or calf stretching, has also been performed [17]. Unfortunately, few studies have specifically explored the predictive validity of possible variables that could be used to identify patients with CAI who could likely succeed with multimodal rehabilitation rather than single rehabilitation techniques.

Therefore, the aim of this study was to identify possible predictors of treatment success following multimodal rehabilitation. The participants’ age, sex, body mass index (BMI), time since last sprain, visual analogue scale (VAS) scores of pain, American Orthopaedic Foot & Ankle Society (AOFAS) scores, Y Balance Test (YBT) performance, drawer test results, and ankle ROM were tested to determine whether these variables have predictive value in determining whether the participants could obtain meaningful results following multimodal rehabilitation.

## Methods

### Study design and participants

We performed a prospective study to identify possible predictors of treatment success following ﻿multimodal rehabilitation. Sixty subjects with CAI (males: 14, females: 46) were recruited between November 2017 and August 2018 from Beijing Tongren Hospital. The inclusion and exclusion criteria were selected according to the criteria endorsed by the International Ankle Consortium [18]. The inclusion criteria for the CAI group were as follows: (1) at least 1 significant unilateral ankle sprain causing inflammatory symptoms and the loss of activity for 1 or more days (the initial sprain occurred more than 12 months prior to the study, and the most recent sprain occurred more than 3 months prior to the study) and (2) a continuing feeling of “instability”, episodes of “giving way” and/or recurrent ankle sprain. The exclusion criteria were as follows: (1) previous lower limb fracture, surgery, or disorders of musculoskeletal structures; (2) systemic diseases or neurological disorders that impact foot and ankle function; (3) generalized joint laxity assessed by the Beighton score [19]; and (4) bilateral ankle sprain or injury history.

This prospective study was approved by the ethics committee of Beijing Tongren Hospital Capital Medical University. The study was performed in accordance with the Declaration of Helsinki. Informed consent was obtained in writing from all participants prior to the study.

### Data collection

All participants provided demographic information, completed an injury history questionnaire and underwent a physical examination. The information collected included details regarding their sex, age, BMI and time since the last ankle sprain, which were collected from the patients’ medical chart. The symptom assessment included pain, swelling, stiffness, instability and repetitive sprain of the injured ankle. The physical signs included ROM restriction and a positive drawer test of the injured ankle. The ROM of the ankle joint was measured using a goniometer with the subject lying in the supine position with the knee bent. The side-to-side difference in the ROM degree was calculated, and deficits greater than 1 degree observed in any direction in dorsiflex, plantarflex, inversion or eversion on the injured side compared to the contralateral healthy side were defined as ROM restriction. The anterior drawer test was performed with the subject seated, knees flexed at 90° and ankles at 10° of plantar flexion. Anterior displacement of the talus relative to the fibula was evaluated subjectively [20, 21]. All participants were evaluated by a pain VAS [22] and the AOFAS score [22]. The VAS score was anchored by “no pain” (score of 0) and “pain as bad as it could be” or “worst imaginable pain” (score of 10 [100-mm scale]) [23].

Dynamic posture control was measured using the YBT (Professional Y Balance Test Kit; Functional Region-Specific Patient-Reported Outcomes Movement Systems, Inc., Chatham, VA) [24]. The participants completed 4 practice trials, followed by 3 test trials in each of the 3 directions. If the participants failed to maintain balance, raised the heel of the stance limb during the test, removed their hands from their hips, used the reach indicator for support, kicked the indicator, or did not return the uninvolved limb to the starting position, the test trials were repeated [25, 26]. The test trials were averaged and normalized to height (%) for the analysis.

### Intervention

Over an 8-week period, each participant received sixteen sessions of 30-min treatment of multimodal rehabilitation in the clinic twice a week. The treatment included ROM, strength, balance, and functional activities. ROM was assessed with four 2-min sets of Maitland grade III mobilization each for anterior-to-posterior talocrural joint mobilization with the patient in the supine position, posterior-to-anterior talocrural joint mobilization with the patient in the prone position, and lateral-to-medial and medial-to-lateral subtalar joint mobilization with the patient in the lateral position. These grade III mobilizations were large-amplitude, approximately 1-s rhythmic oscillations from the mid-to-end ROM to tissue resistance [2]. Strength training was performed using TheraBand resistance training for dorsiflexion, plantar flexion, inversion and eversion muscles, with 10 repetitions each lasting 10 s, 2 sessions per direction. The rest time between sessions was 2 min. The participants sat on the floor and extended their knees, with one end of the elastic band attached to a table and the other end attached to the metatarsal head of the involved foot. The band was stretched to an additional 70% of its resting length. The participants were instructed to slowly contract concentrically for 3 s, hold at the end range of motion for 6 s, and slowly contract eccentrically for 3 s. The female participants used a red medium-resistance band during the first four weeks and a green heavy-resistance band during the last four weeks. The male participants used a green heavy resistance band during the first four weeks and a blue extraheavy resistance band during the last four weeks. Then, the participants were asked to perform 10 repetitions, each lasting 10 s, bipedal calf raise for 2 min. Balance training was performed with opened eyes using a BOSU® ball, first in the double-leg stand position as long as they could until 30 s. When the participants could stand on the BOSU® ball with double legs for 30 s, they were instructed to stand on the BOSU® ball in the single-leg stand position. In each session, the participants were requested to remain in the single-leg stand position for as long as they could until 30 s. During the balance exercise, their hands should remain on their hips. In total, 3–5 sessions of BOSU® ball exercise were performed according to the patient’s endurance. The functional tasks included 3 sessions of 1-min gait training or 2 sessions of 30-s box hops according to the patient’s function. Gait training was performed by a supervised walk on the floor and verbal feedback based on the therapist’s observation. When the participants could walk without pain and no inversion or eversion of the hindfoot was observed by the therapist, a box hop was selected. The participants were asked to perform a single leg vertical drop from a 40 cm high box. They were instructed to step down and maintain balance with the injured foot. Four to 6 single leg vertical drops were performed in one session. The therapist watched and provided verbal feedback. Home exercises included three 30-s sets of gastrocnemius-soleus complex stretching, with the stand and knee straight. Ankle strengthening in four directions was the same as that performed in the clinic. The participants were provided TheraBand to take home. The participants performed home exercise 5 times per week.

### Follow-up

Of the 60 patients with CAI who met the study inclusion criteria, 51 patients (85.0%) were available for follow-up within 1 month after 8 weeks of the intervention through telephone investigation (males: 10, females: 41). The participants were dichotomized as success or nonsuccess based on the treatment response as indicated by the GRC. Participants selecting any of the nine responses ranging from “a very great deal worse” to “somewhat better” were dichotomously categorized as unsuccessful, whereas those selecting the four responses ranging from “moderately better” to “a very great deal better” were categorized as successful [27].

### Statistical analysis

We used SPSS 26.0 (IBM Co., Inc., Chicago, IL, USA) and MedCalc 20.0.3 (MedCalc, Mariakerke, Belgium) for the statistical analyses. The Kolmogorov–Smirnov test was used for normality testing. The continuous data are expressed as the mean ± standard deviation (SD). We used independent samples t tests to analyse the continuous variables (e.g., age) and chi-squared tests to analyse the categorical variables (e.g., sex) and compare the differences between the successful and unsuccessful groups. Variables with a significance level of *p* ≤ 0.10 were retained as potential predictor variables [17]. A stepwise logistic regression model was then used to determine the most accurate set of potential predictor variables for predicting the success of rehabilitation. Variables with a significance level of *p* > 0.10 were removed from the equation to further reduce the risk of excluding variables that might help strengthen the model.

All potential predictor variables were subjected to a receiver operator characteristic (ROC) curve analysis [14]. The sensitivity, specificity, and positive and negative likelihood ratios (LRs) were calculated to identify cut-off scores for each potential predictor variable. The diagnostic accuracy and probability of success were also calculated for a combination of predictor variables. Youden’s index was used to identify cut-off scores. + LR was used to calculate the posttest probability of treatment success. The pretest probability was assumed to be equal to the proportion of patients who were categorized as successful.

## Results

After completing the rehabilitation sessions, 40 of 51 participants (78.4%) were considered to have a successful outcome. The univariate comparisons of the potential predictor variables between the successful and unsuccessful groups sorted by treatment group are shown in Table 1.

**Table 1:** Univariate comparisons of the successful and unsuccessful groups

Variable	Successful	Unsuccessful	*P*
	(*n* = 40)	(*n* = 10)	
Age, y	30.65 $\pm$ 10.63	37.55 $\pm$ 11.18	0.065 ^a^
Sex: female, n (%)	33(82.5%)	8(72.7%)	0.769
BMI, kg/m^2^	22.30 $\pm$ 3.67	24.10 $\pm 3.90$	0.159
Time since last sprain (months)	12.50 $\pm$ 17.36	29.82 $\pm$ 29.23	0.027 ^a^
VAS score	4.35 $\pm$ 2.01	4.68 $\pm$ 2.69	0.548
AOFAS score	75.34 $\pm$ 14.44	82.82 $\pm$ 7.48	0.107
YBT-A (%height)	34.03 $\pm$ 6.19	35.50 $\pm$ 7.16	0.488
YBT-PL (%height)	34.11 $\pm$ 5.58	32.80 $\pm$ 7.16	0.541
YBT-PM (%height)	36.09 $\pm$ 5.73	34.80 $\pm$ 5.96	0.538
Positive drawer test, n (%)	11(27.5%)	4(36.4%)	0.843
Restrictive ankle ROM, n (%)	32(80.0%)	10(90.9%)	0.694

*BMI* Body mass index, *VAS* Visual analogue scale, *AOFAS* American Orthopaedic Foot & Ankle Society, *YBT-A* Y balance test anterior reach, *YBT-PL* Y balance test posterolateral reach, *YBT-PM* Y balance test posteromedial reach, *ROM* Range of motion

^a^ Indicates a significant difference (*p* ≤ 0.10) between the successful and unsuccessful groups

The univariate variables that were retained after the stepwise regression of treatment success are shown in Table 2. The variable in the regression analysis was time since the last sprain. The posttest probability was 88.03, indicating that individuals with a time since last sprain ≤ 8 months had an 88.03% chance of a successful outcome. The positive LR of this model indicates small but sometimes important shifts in probability of treatment success [28]. Although an age > 36 years had a higher posttest probability, it could not be eliminated in the stepwise regression.

**Table 2:** Sensitivity, specificity, positive likelihood ratio, posttest probability, and odds ratios of individual predictor variables

	Sensitivity, % (95% CI)	Specificity, % (95% CI)	Positive likelihood ratio (95% CI)	Posttest probability %	Odds ratio (95% CI)
Time since last sprain > 8 months ^a^	72.73(39.0, 94.0)	65.00(48.3, 79.4)	2.08(1.2, 3.6)	88.03	4.95(1.13, 21.70)
Age > 36 y	72.73(39.0, 94.0)	80.0(64.4, 90.9)	3.64(1.8, 7.5)	92.96	7.03(1.57, 31.43)

*CI* Confidence interval

^a^ Retained in the regression model

## Discussion

The most important finding of this study is that there is a specific predictor variable that can significantly improve the prediction of successful outcomes in patients with CAI who underwent multimodal rehabilitation. Specifically, we found that the time since the last sprain can be used to predict successful outcomes following multimodal rehabilitation programs. The pretest probability was 78.4%, and patients with a time since the last sprain of ≤ 8 months were more likely to have a meaningful outcome in the global sense of improvement; the posttest probability was 88.03%. The current data suggest that 78.4% of patients could have successful outcomes after this impairment-based progressive rehabilitation process. If the criterion of the time since the last ankle sprain ≤ 8 months was met, 88.03% of the patients had successful outcomes after this rehabilitation process. However, there was only a 9.63% increase in the probability of successful treatment. This predictor could help identify patients who may have successful outcomes before treatment.

Previous CAI secondary analyses have reported measurements by the self-reported function Foot and Ankle Ability Measure–Sport (FAAM–S). Less than 5 recurrent ankle sprains and a Foot and Ankle Ability Measure score below 82.7% are likely indicators of success with ankle joint mobilization [2]. An age below 22 years and a weight-bearing lunge test below 9.9 cm are likely indicators of success with plantar massage [2]. Similarly, in our findings, the age of the successful group was younger than that of the unsuccessful group. Measured by the single-limb balance test (SLBT), SLBT errors ≥ 3 and FAAM between limb differences > 11.3% are likely indicators of success with ankle joint mobilization, and SLBT ≥ 3 errors and FAAM between limb differences < 16.07% are likely indicators of success with plantar massage [17]. These two studies were secondary investigations, and the sample sizes were small, but they provide evidence for clinicians treating patients with CAI. In another secondary analysis using data from 6 previous investigations, Burcal et al. [14] found that Star Excursion Balance Test Posteromedial (SEBT-PM) reach distance ≤ 85.18% and self-reported function activities of daily living score ≤ 92.55% were significant predictors of improvement in SEBT-PM after balance training. However, in our study, there was no significant difference in the SEBT score between the two groups. Previous studies did not investigate indicators of the comprehensive treatment of ROM, strength, balance, and functional activities. Patients with CAI may have multiple deficits, and the 4 domains of ROM, strength, balance, and functional activities were suggested by Donovan and Hertel [10]. For patients with a prognosis related to BMI, Rosen et al. [29] recently conducted a pooled multisite analysis and reported that patients with multiple ankle sprains had a higher BMI and self-reported disability than those with a single sprain. In our study, the unsuccessful group also had a slightly higher BMI, although the difference was not statistically significant. The VAS scores of the two groups showed no significant difference. The average VAS score was approximately 4, which may have limited the patients’ daily activities and led the patients to visit our clinic. The AOFAS score of the successful group was slightly higher than that of the unsuccessful group, although the difference was not statistically significant. A positive drawer test and restrictive ankle ROM did not significantly influence the patient-reported outcome.

In this study, we collected patient characteristics, VAS for pain rating data, AOFAS for functional scale, YBT for dynamic balance test, drawer test for joint laxity, and ROM restriction to detect the potential indicators of successful impairment-based rehabilitation, but only age and time since last ankle sprain showed significant differences, and time since last ankle sprain was retained in the regression model. The reason why people with a more recent acute ankle sprain are more likely to demonstrate meaningful improvements may be because their movement modality was easier to correct. In future studies, we should record more subjective and objective evaluations before and after the intervention to provide more evidence for potential predictions.

There are limitations in this study. First, we used perceived global improvement in self-reported outcomes. After 8 weeks, the participants might not remember the level of function they had prior to the study, which may generate bias [30]. In further studies, more quantitative self-assessment scales or objective evaluations may add to the identification of indicators of treatment success. Second, we used height instead of leg length to normalize the YBT excursion distance, which may be less accurate. Third, although all participants completed the intervention in the clinic, we did not consider their home exercise compliance. These findings may be improved in future studies.

## Conclusion

This preliminary study demonstrates that certain patient characteristics can predict whether a participant with CAI will have successful outcomes after receiving multimodal rehabilitation. In particular, patients who had a time since the last sprain of less than 8 months were most likely to have a successful result. These results may serve as a basis for further studies and assist clinical decision making in the treatment of patients with CAI using multimodal rehabilitation.

## Supplementary Information

**Additional file 1: Appendix 1.** Multimodal rehabilitation in the clinic and home exercises.

## Data Availability

The datasets used in the current study are available from the corresponding author upon reasonable request.

## Abbreviations

CAI: Chronic ankle instability
GRC: Global rating of change
LAS: Lateral ankle sprain
ROM: Range of motion
BMI: Body mass index
VAS: Visual analogue scale
AOFAS: American Orthopaedic Foot & Ankle Society
YBT: Y Balance Test
ROC: Receiver operator characteristic
LRs: Likelihood ratios
FAAM: Foot and ankle ability measure
SEBT: Star Excursion Balance Test
