# Supplementary material for: Predicting the success of multimodal rehabilitation in chronic ankle instability based on patient-reported outcomes
Source: BMC Musculoskelet Disord. 2022 Jul 25;23:706. doi: 10.1186/s12891-022-05676-0 (PMC9310506; doi:10.1186/s12891-022-05676-0)
Supplement: Supplementary file 1 — Additional file 1: Appendix 1. Multimodal rehabilitation in the clinic and home exercises. [file 12891_2022_5676_MOESM1_ESM.docx]

Appendix 1

Multimodal rehabilitation in the clinic and home exercises

| Intervention | Description |
| --- | --- |
| Multimodal rehabilitation in the clinic |  |
| ROM | Four 2-minute sets of ﻿Maitland grade III mobilization each for anterior-to-posterior and posterior-to-anterior talocrural joint mobilization and lateral-to-medial and medial-to-lateral subtalar joint mobilization. |
| Strength training | TheraBand resistance training for dorsiflexion, plantar flexion, inversion and eversion muscles, ﻿with 10 repetitions each lasting 10 seconds, 2 sessions per direction. Rest between sessions was 2 minutes. |
| Balance training | Using a BOSU﻿® ball, first, the participants held the double-leg stand position for as long as they could until 30 seconds. Then, the participants stood on a BOSU﻿® ball in the single-leg stand position for as long as they could until 30 seconds. A total of 3-5 sessions, all with opened eyes. |
| Gait training | 3 sessions of 1-minute supervised gait training; the participants walked on the floor and received verbal feedback based on the therapist’s observation. |
| Box hop | 2 sessions of 30-second box hops, the participants performed a single leg vertical drop from a 40 cm high box, stepped down and maintained balance with the injured foot; 4 to 6 single leg vertical drops per session. The therapist watched and gave verbal feedback. |
| Home exercise |  |
| Stretching | ﻿Three 30-second sets of ﻿gastrocnemius-soleus complex stretching, while standing with the knee straight. |
| Strength training | TheraBand resistance training for dorsiflexion, plantar flexion, inversion and eversion muscles, ﻿with 10 repetitions each lasting 10 seconds, 2 sessions per direction. Rest between sessions was 2 minutes. |
